# 500 minimalinvasive Leberresektionen – Erfahrungen, Ergebnisse und technische Entwicklungen eines High-Volume-Zentrums

**DOI:** 10.1007/s00104-025-02373-1

**Published:** 2025-09-06

**Authors:** Schaima Abdelhadi, Mohamad El-Ahmar, Flavius Sandra-Petrescu, Christoph Reissfelder

**Affiliations:** 1 Chirurgische Klinik der Universitätsmedizin Mannheim, Medizinische Fakultät Mannheim der Universität Heidelberg, Mannheim, Theodor-Kutzer-Ufer 1–3, 68167 Mannheim, Deutschland; 2 DKFZ-Hector Krebsinstitut an der Universitätsmedizin Mannheim, Mannheim, Deutschland

**Keywords:** Hepatektomie, Laparoskopische Leberresektion, Roboterassistierte Leberresektion, Perioperative Ergebnisse, Hepatectomy, Laparoscopic liver resection, Robotic-assisted liver resection, Perioperative outcomes

## Abstract

**Hintergrund:**

Die minimalinvasive Leberchirurgie hat sich in den vergangenen Jahren dynamisch weiterentwickelt. Neben der laparoskopischen Leberresektion (LLR) gewinnt die roboterassistierte Leberresektion (RLR) zunehmend an Bedeutung. Ob die roboterassistierte Technik insbesondere bei komplexen Resektionen klinische Vorteile bietet, wird derzeit noch kontrovers diskutiert.

**Ziel:**

Ziel dieser Arbeit war es, die Entwicklung, perioperative Ergebnisse sowie zentrale Herausforderungen und Erfahrungswerte aus über 500 minimalinvasiven Leberresektionen an einem spezialisierten High-Volume-Zentrum darzustellen. Im Fokus steht der Vergleich zwischen LLR und RLR unter Berücksichtigung des IWATE-Schwierigkeitsgrads.

**Material und Methoden:**

Es erfolgte eine retrospektive monozentrische Analyse von 526 konsekutiven elektiven minimalinvasiven Leberresektionen (2018–2024), stratifiziert nach IWATE-Score. Verglichen wurden LLR und RLR hinsichtlich operativer Parameter, Konversionsrate und postoperativer Komplikationen. Zusätzlich wurde die jährliche Entwicklung der Verfahren analysiert.

**Ergebnisse:**

Die RLR wurde ab 2021 etabliert und machte im Jahr 2024 bereits über 50 % der minimalinvasiven Resektionen aus. Im Vergleich zur LLR war RLR mit einem signifikant geringeren intraoperativen Blutverlust sowie niedrigeren Konversions- und Komplikationsraten assoziiert, insbesondere bei technisch anspruchsvollen Resektionen. Trotz eines steigenden Anteils an Advanced/Expert-Resektionen konnte die Rate schwerwiegender Komplikationen im Zeitverlauf gesenkt werden.

**Schlussfolgerung:**

Minimalinvasive Leberresektionen sind an High-Volume-Zentren sicher durchführbar. Die roboterassistierte Technik bietet insbesondere bei komplexen Resektionen Vorteile hinsichtlich Komplikationsrate, Konversion und Blutverlust. Ein zentraler Erfolgsfaktor für die Einführung der robotischen Leberchirurgie ist die bereits vorhandene Expertise in der laparoskopischen Technik, durch die sich die Lernkurve erheblich verkürzen ließ. Der Einsatz standardisierter Techniken wie der „Scissor Hepatectomy“ führte möglicherweise zu einer vergleichsweise niedrigen Rate an Galleleckagen im Rahmen der RLR.

**Graphic abstract:**

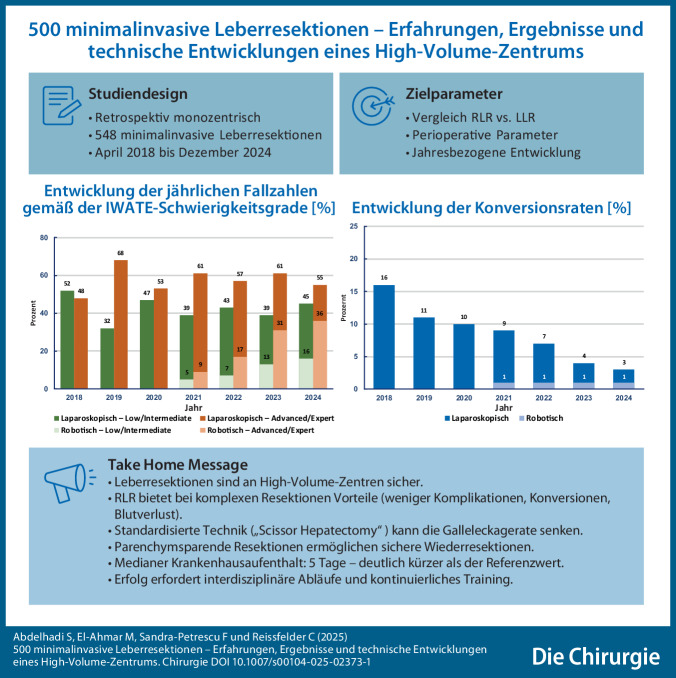

**Zusatzmaterial online:**

Die Online-Version dieses Beitrags (10.1007/s00104-025-02373-1) enthält weitere Tabellen.

## Abstract (en)

**Background:**

Minimally invasive liver surgery has rapidly evolved in recent years. In addition to the laparoscopic liver resection (LLR), robot-assisted liver resection (RLR) is increasingly gaining in importance; however, although the robotic-assisted approach offers clinical benefits, particularly in complex procedures, it remains a matter of debate.

**Objective:**

The aim of this study was to present the development, perioperative outcomes, key challenges, and insights from over 500 minimally invasive liver resections performed at a specialized high-volume center. It focused on the comparison between LLR and RLR based on the IWATE difficulty score.

**Material and methods:**

A retrospective single-center analysis of 526 consecutive elective minimally invasive liver resections (2018–2024) was conducted. All cases were stratified using the IWATE score. The LLR and RLR were compared in terms of operative parameters, conversion rate and postoperative complications. Additionally, the annual procedural development was analyzed.

**Results:**

The RLR was established in 2021 and accounted for over 50% of all minimally invasive liver resections by 2024. Compared to LLR, RLR was associated with significantly reduced intraoperative blood loss as well as lower conversion and complication rates, particularly in technically demanding resections. Despite an increasing proportion of advanced/expert resections, the rate of major complications could be reduced over time.

**Conclusion:**

Minimally invasive liver resections can be safely performed at high-volume centers. The robotic-assisted technique offers specific advantages especially in complex resections, with respect to complication rates, reduced conversion rates and decreased blood loss. A key success factor in the implementation of robotic liver surgery is the pre-existing expertise in laparoscopic techniques, which significantly shortened the learning curve. The use of standardized techniques such as the scissor hepatectomy may have contributed to the comparatively low rate of bile leaks observed in RLR.

## Hintergrund und Fragestellung

Die minimalinvasive Leberchirurgie (MILR) hat sich in den vergangenen Jahrzehnten zu einem etablierten Standardverfahren in der hepatobiliären Chirurgie entwickelt [1]. Technologische Fortschritte in der Bildgebung, die Weiterentwicklung chirurgischer Instrumente sowie die Einführung standardisierter Operationsverfahren haben wesentlich dazu beigetragen, dass heute auch komplexe Leberresektionen zunehmend minimalinvasiv durchgeführt werden können [1]. Zahlreiche Studien zeigen, dass die laparoskopische Leberresektion (LLR) im Vergleich zur offenen Leberresektion (OLR) mit einem geringeren intraoperativen Blutverlust, einer kürzeren Krankenhausverweildauer sowie einer niedrigeren postoperativen Morbidität assoziiert ist [1–3].

Dennoch stellt die Leberchirurgie weiterhin eine der technisch anspruchsvollsten Disziplinen innerhalb der minimalinvasiven Chirurgie dar. Insbesondere die Präparation in Gefäßnähe, die intraoperative Navigation ohne direkte haptische Kontrolle und das Komplikationsmanagement erfordern ein hohes Maß an chirurgischer Expertise, standardisierte Abläufe und eine enge interdisziplinäre Zusammenarbeit [4]. Trotz der genannten Vorteile unterliegt die konventionelle LLR weiterhin technischen Limitationen. Dazu zählen die eingeschränkte Beweglichkeit der Instrumente, die präzise Trokarposition sowie ergonomisch herausfordernde Arbeitsbedingungen – insbesondere bei komplexeren Resektionen [5, 6].

Vor diesem Hintergrund hat die roboterassistierte Leberresektion (RLR) in den vergangenen Jahren zunehmend an Bedeutung gewonnen. Robotische Operationssysteme bieten insbesondere durch artikulierte Instrumente mit erweiterten Freiheitsgraden potenzielle technische Vorteile gegenüber der konventionellen LLR [7–9].

Ziel dieser Arbeit war es, die Entwicklung, perioperativen Ergebnisse sowie die wichtigsten Herausforderungen und Erfahrungswerte aus über 500 minimalinvasiven Leberresektionen an einem spezialisierten High-Volume-Zentrum darzustellen. Im Fokus steht dabei der Vergleich zwischen der LLR und der RLR.

## Methoden

### Studiendesign und Patientenkohorte

In diese retrospektive Analyse wurden alle konsekutiven Patienten eingeschlossen, die zwischen April 2018 und Dezember 2024 am Universitätsklinikum Mannheim, Medizinische Fakultät Mannheim der Universität Heidelberg, eine elektive Leberresektion erhielten. Die Datenerhebung erfolgte auf Grundlage einer prospektiv geführten institutseigenen Datenbank.

Einschlusskriterien waren ein Alter von mindestens 18 Jahren sowie die Durchführung einer elektiven minimalinvasiven Leberresektion. Notfalloperationen und multiviszerale Resektionen wurden ausgeschlossen. Die Studie wurde von der Ethikkommission der Universität Heidelberg genehmigt (AZ: 2024-839). Eine Registrierung erfolgte im Deutschen Register für Klinische Studien (DRKS00036636).

### Definitionen

Leberresektionen wurden gemäß der Brisbane-2000-Klassifikation eingeteilt. Anatomische Resektionen wurden entsprechend der Couinaud-Segmentierung als vollständige Entfernung eines oder mehrerer portalvenöser Segmente definiert. Zur Bewertung der technischen Schwierigkeit der Eingriffe wurde der IWATE-Score verwendet [10]. Der Score berücksichtigt 6 Parameter: Tumorlokalisation, Ausmaß der Resektion, Tumorgröße, Nähe zu großen Gefäßen, das verwendete Verfahren (konventionell laparoskopisch vs. handassistiert [HALS] oder Hybridtechnik) sowie die Leberfunktion (Child-Pugh A/B). Die resultierende Punktzahl (0–12) erlaubt eine Einteilung der Resektionen in 4 Schwierigkeitsgrade („low“, „intermediate“, „advanced“ und „expert“). In der vorliegenden Arbeit wurden zur besseren Vergleichbarkeit 2 Gruppen zusammengefasst: Low/Intermediate (0 bis 6 Punkte) und Advanced/Expert (7 bis 12 Punkte).

Patienten, bei denen eine Konversion von laparoskopischem oder roboterassistiertem Vorgehen auf eine offene Resektion erforderlich war, wurden nach dem Prinzip der Intention-to-treat-Analyse dem ursprünglich geplanten Vorgehen zugeordnet.

Postoperative Komplikationen wurden gemäß der Clavien-Dindo-Klassifikation eingeteilt [11]. Leberspezifische Komplikationen wurden gemäß den Definitionen der International Study Group of Liver Surgery (ISGLS) erfasst, berücksichtigt wurden ausschließlich Komplikationen des Grades B und C [12, 13].

Primärer Endpunkt war das Auftreten postoperativer Komplikationen innerhalb von 90 Tagen nach der Operation.

### Standardisierte perioperative Versorgung

Alle Patienten wurden prä-, intra- und postoperativ gemäß standardisierter institutsinterner Behandlungsprotokolle betreut. Bei onkologischen Indikationen erfolgte die präoperative Fallbesprechung in einer interdisziplinären Tumorkonferenz. Die Eingriffe wurden ausschließlich durch 3 erfahrene hepatobiliäre Chirurgen durchgeführt.

### Operatives Vorgehen

Die Durchführung der LLR und RLR erfolgte gemäß einem standardisierten operativen Vorgehen durchgeführt, das bereits in früheren Arbeiten beschrieben wurde [14–16]. Die Patienten wurden in Rückenlagerung in Reverse-Trendelenburg-Position gelagert. Nach Etablierung eines Pneumoperitoneums mit einem intraabdominellen Druck von 12 mm Hg erfolgte eine intraoperative Sonographie zur Beurteilung der Resektabilität und Festlegung der Resektionslinien. Bei LLR erfolgte die Parenchymdissektion unter Anwendung der bipolaren Zange und der Crush-Clamp-Technik in Kombination mit Versiegelungsinstrumenten (LigaSure™, Medtronic, Minneapolis, MN, USA; Thunderbeat™, Olympus Medical Systems Corp., Tokyo, Japan). Bei RLR wurde die Parenchymdissektion unter Verwendung der monopolaren Schere auf der Da Vinci Xi- oder X‑Plattform (Intuitive Surgical, Sunnyvale, CA, USA) durchgeführt [17]. In beiden Gruppen wurden intrahepatische Gefäße je nach Durchmesser und Lokalisation mittels linearer Stapler, Hem-o-lok-Clips oder Titanclips durchtrennt. Ein intermittierendes Pringle-Manöver wurde bei Bedarf mittels eines um das Ligamentum hepatoduodenale gelegten Foley-Katheters durchgeführt. Eine routinemäßige Lymphadenektomie erfolgte ausschließlich bei Verdacht auf ein cholangiozelluläres Karzinom.

Das Resektionspräparat wurde entweder über einen Pfannenstiel-Schnitt oder über die Eröffnung vorbestehender Narben geborgen. Die Anlage intraabdomineller Drainagen erfolgte nicht routinemäßig.

### Statistische Analyse

Die statistische Auswertung erfolgte mit der Statistiksoftware Jamovi (Version 2.6.26; The Jamovi Project, Sydney, Australien). Kategoriale Variablen wurden als absolute und relative Häufigkeiten dargestellt und mit dem Chi^2^-Test oder Fisher’s Exact-Test verglichen. Stetige Variablen wurden je nach Verteilung als Mittelwert ± Standardabweichung oder als Median mit Interquartilsabstand angegeben. Gruppenvergleiche erfolgten mittels Student’s t‑Test (bei normalverteilten Daten) oder Mann-Whitney-U-Test (bei nichtparametrischer Verteilung). Ein *p*-Wert < 0,05 wurde als statistisch signifikant gewertet.

## Ergebnisse

**Abb. 1:**
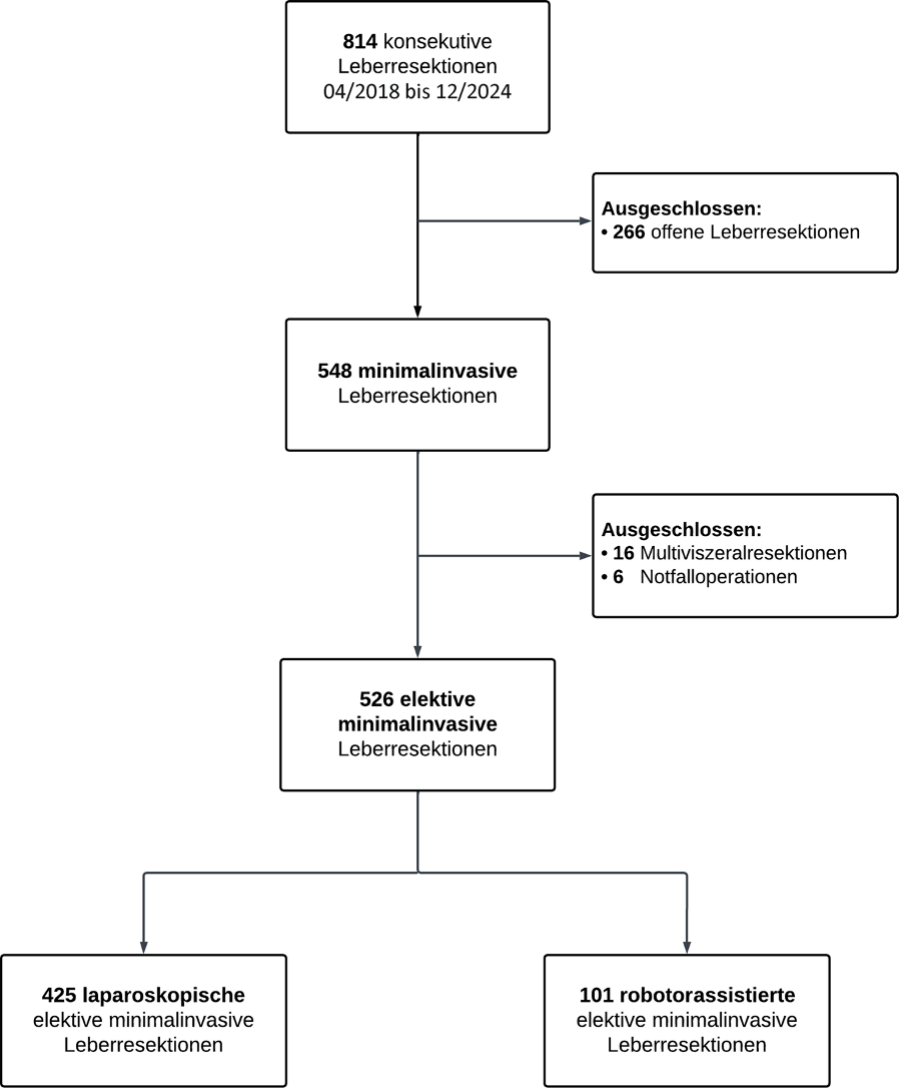
Flowchart der Studie

Im Zeitraum von April 2018 bis Dezember 2024 wurden an unserem Zentrum insgesamt 814 konsekutive Leberresektionen durchgeführt. Davon erfolgten 548 Eingriffe (67 %) in minimalinvasiver Technik. Nach Ausschluss von 6 Notfalloperationen und 16 multiviszeralen Resektionen wurden 526 elektive minimalinvasive Leberresektionen in die finale Analyse eingeschlossen. Davon wurden 425 Resektionen (81 %) laparoskopisch und 101 Resektionen (19 %) roboterassistiert durchgeführt **(**Abb. 1**)**.

### Patientencharakteristika

Die demografischen und klinischen Patientencharakteristika waren zwischen beiden Gruppen weitgehend vergleichbar (Tab. 1). Das mediane Alter betrug 65 Jahre in der LLR-Gruppe und 67 Jahre in der RLR-Gruppe. Der BMI war in der RLR-Gruppe signifikant niedriger (25 vs. 26 kg/m^2^, *p* = 0,01), während der Anteil männlicher Patienten in der LLR-Gruppe höher war (58 % vs. 47 %, *p* = 0,04). Weitere Parameter wie die ASA-Klassifikation, das Vorliegen von Komorbiditäten, frühere Vorbehandlungen oder eine Leberzirrhose zeigten keine signifikanten Unterschiede zwischen den Gruppen.

**Tab. 1:** Patientencharakteristika der Studienkohorte

	LLR *n* = 425	RLR *n* = 101	*p*-Wert
**Alter**, Jahre^a^	65 (56–74)	67 (56–77)	0,47
**BMI**, kg/m^2a^	26 (23–30)	25 (22–28)	**0,01**
**Geschlechterverteilung** (m:w)	246:179	47:54	**0,04**
**ASA-Klassifikation**^b^			0,45
*I*	20 (4)	6 (6)	–
*II*	215 (51)	41 (41)	–
*III*	183 (43)	52 (51)	–
*IV*	7 (2)	2 (2)	–
**Kardiovaskuläre Vorerkrankungen**^b^	237 (56)	49 (49)	0,62
**Diabetes mellitus**^b^	94 (22)	20 (20)	0,57
**Pulmonale Vorerkrankungen**^b^	66 (16)	16 (16)	0,67
**Leberzirrhose**^b^	54 (13)	13 (13)	0,70
*Child A*	46 (11)	10 (10)	–
*Child B*	8 (2)	3 (3)	–
**Ätiologie der Leberzirrhose**^b^			0,47
*Äthyltoxisch*	27 (6)	6 (6)	–
*Viral*	17 (4)	5 (5)	–
Hepatitis B	11 (3)	3 (3)	–
Hepatitis C	6 (1)	2 (2)	–
*MASLD*	10 (2)	2 (2)	–
**Vorbehandlungen**^b^			
*Vorangegangene Leberresektion*	80 (19)	10 (10)	0,06
*Vorangegangene lokalregionale Therapie*	17 (4)	2 (2)	0,40
*Vorangegangene systemische Therapie*	60 (14)	11 (11)	0,58
**Diagnose**^b^			0,19
*Primär maligne Lebertumoren*	135 (32)	32 (32)	–
HCC	90 (21)	20 (20)	–
CCC	42 (10)	11 (11)	–
GBC	3 (1)	1 (1)	–
*Lebermetastasen*	188 (44)	42 (42)	–
Anzahl der Metastasen^c^	3 ± 3	2 ± 3	–
*Benigne Lebertumoren*	102 (24)	27 (26)	–

*BMI* Body-Mass-Index, *ASA* American Society of Anesthesiologists, *MASLD* metabolisch-assoziierte Steatose-Lebererkrankung, *HCC* hepatozelluläres Karzinom, *CCC* cholangiozelluläres Karzinom, *GBC* Gallenblasenkarzinom

^a^Werte als Median (Interquartilsabstand)

^b^Werte als absolute Zahlen (Prozentwert)

^c^Werte als Mittelwert ± Standardabweichung

Am häufigsten lag eine Lebermetastasierung als Operationsindikation vor (LLR: 44 %, RLR: 42 %); die durchschnittliche Anzahl der Metastasen betrug dabei 3 ± 3 in der LLR-Gruppe und 2 ± 3 in der RLR-Gruppe. Seltener erfolgte die Resektion aufgrund primär maligner Lebertumoren (LLR: 32 %, RLR: 32 %) oder benigner Leberläsionen (LLR: 24 %, RLR: 26 %).

### Operative Parameter und postoperative Ergebnisse

Die Studienkohorte wurde gemäß dem IWATE-Score in 2 Subgruppen stratifiziert, um Operationscharakteristika und perioperative Ergebnisse in Abhängigkeit vom Schwierigkeitsgrad der Resektion zu analysieren. Dabei wurden Low/Intermediate-Resektionen und Advanced/Expert-Resektionen unterschieden. Die entsprechenden Ergebnisse sind in Tab. 2 und 3 zusammengefasst.

**Tab. 2:** Operative Parameter und postoperative Ergebnisse bei Advanced/Expert-Leberresektionen

	LLR *n* = 232	RLR *n* = 76	*p*-Wert
*Operationsverfahren*^*a*^			0,09
Atypische Leberresektion	70 (30)	20 (26)	–
Anzahl atypischer Resektionen^b^	2 ± 1	2 ± 1	0,78
(Erweiterte) Hemihepatektomie rechts	56 (24)	17 (22)	–
(Erweiterte) Hemihepatektomie links	27 (12)	7 (9)	–
Linke laterale Sektorektomie	8 (3)	2 (3)	–
Rechts posteriore Sektorektomie	14 (6)	4 (5)	–
Rechts anteriore Sektorektomie	1 (1)	5 (7)	–
Sonstige Mono- oder Bisegmentresektionen	90 (39)	24 (31)	–
*Konversion*^a^	29 (12)	3 (4)	**0,03**
*Operationszeit*, min	261 (194–355)	320 (246–404)	**0,005**
*Pringle-Manöver*	136 (59)	60 (79)	0,19
Dauer, min	34 (19–62)	56 (33–92)	**<** **0,001**
*Blutverlust,* ml^c^	650 (300–1400)	500 (288–1100)	**0,03**
*Postoperative Komplikationen*^d^	101 (44)	16 (21)	**0,03**
Grad I	37 (16)	4 (5)	***–***
Grad II	27 (10)	5 (7)	***–***
Grad III	20 (9)	3 (4)	***–***
Grad IV	7 (3)	1 (1)	***–***
Grad V	10 (4)	3 (4)	***–***
*Art der Komplikationen*^a^			
Wundinfektion	10 (4)	1 (1)	0,41
Platzbauch	5 (2)	0 (0)	0,31
Pleuraerguss mit Atelektasen	16 (7)	2 (3)	0,51
Lungenembolie	4 (2)	2 (3)	0,06
Posthepatektomie-Leberblutung^e^	6 (3)	1 (1)	0,57
Posthepatektomie-Gallenleckage^e^	18 (8)	4 (5)	0,54
Posthepatektomie-Leberversagen^e^	13 (6)	2 (3)	0,34
*Krankenhausverweildauer*, Tage^c^	6 (5–10)	6 (4–9)	0,34

^a^Werte als absolute Zahlen (Prozentwert)

^b^Werte als Mittelwert ± Standardabweichung

^c^Werte als Median (Interquartilsabstand)

^d^Klassifikation nach Clavien-Dindo

^e^Klassifikation gemäß ISGLS; ausschließlich Grad B und C berücksichtigt

**Tab. 3:** Operative Parameter und postoperative Ergebnisse bei Low/Intermediate-Leberresektionen

	LLR *n* = 193	RLR *n* = 25	*p-*Wert
*Operationsverfahren*^*a*^			0,67
Atypische Leberresektion	107 (55)	12 (48)	–
Anzahl atypischer Resektionen^b^	2 ± 1	1 ± 1	0,54
Linke laterale Sektorektomie	45 (23)	6 (24)	–
Sonstige Mono- oder Bisegmentresektionen	41 (21)	7 (28)	–
*Konversion*^a^	13 (7)	1 (4)	0,82
*Operationszeit*, min	119 (77–180)	180 (122–237)	**0,003**
*Pringle-Manöver*^a^	59 (30)	10 (40)	0,33
Dauer, min	24 (15–42)	23 (17–34)	0,33
*Blutverlus*t, ml^c^	100 (10–300)	150 (50–300)	0,48
*Postoperative Komplikationen*^*d*^	39 (20)	3 (12)	0,66
Grad I	14 (7)	1 (4)	–
Grad II	6 (3)	1 (4)	–
Grad III	13 (7)	0 (0)	–
Grad IV	3 (1)	0 (0)	***–***
Grad V	3 (1)	1 (4)	***–***
*Art der Komplikationen*^*a*^			
Wundinfektion	1 (1)	0 (0)	0,72
Platzbauch	1 (1)	0 (0)	0,72
Pleuraerguss mit Atelektasen	5 (2)	1 (4)	0,09
Lungenembolie	0 (0)	0 (0)	–
Posthepatektomie-Leberblutung^e^	2 (1)	0 (0)	0,61
Posthepatektomie Gallenleckage^e^	7 (4)	0 (0)	0,33
Posthepatektomie-Leberversagen^e^	0 (0)	0 (0)	–
*Krankenhausverweildauer*, Tage^c^	4 (3–6)	4 (4–5)	0,63

^a^Werte als absolute Zahlen (Prozentwert)

^b^Werte als Mittelwert ± Standardabweichung

^c^Werte als Median (Interquartilsabstand)

^d^Klassifikation nach Clavien-Dindo

^e^Klassifikation gemäß ISGLS; ausschließlich Grad B und C berücksichtigt

Unabhängig vom Schwierigkeitsgrad dominierten parenchymsparende Resektionsverfahren wie atypische Resektionen sowie Mono- und Bisegmentektomien, die insgesamt über 65 % aller Eingriffe ausmachten. Die durchschnittliche Anzahl atypischer Resektionen pro Eingriff betrug bei Advanced/Expert-Resektionen in beiden Gruppen 2 ± 1, in der Low/Intermediate-Subgruppe lag sie bei 2 ± 1 (LLR) bzw. 1 ± 1 (RLR). In beiden Gruppen wurden im Rahmen eines minimalinvasiven Eingriffs maximal 4 atypische Resektionen pro Eingriff durchgeführt. Eine höhere Anzahl von Resektionen – darunter 2 Eingriffe mit jeweils 5 und ein Eingriff mit 7 atypischen Resektionen – erfolgte ausschließlich nach Konversion zur offenen Operation.

### Advanced/Expert-Leberresektionen

In der Subgruppe der Advanced/Expert-Leberresektionen (*n* = 308) wurden 232 Resektionen laparoskopisch und 76 roboterassistiert durchgeführt. Die mediane Operationszeit war in der RLR-Gruppe signifikant länger (320 min [246–404] vs. 261 min [194–355], *p* = 0,005), während der intraoperative Blutverlust signifikant geringer war (500 ml [288–1100] vs. 650 ml [300–1400], *p* = 0,03). Die Konversionsrate war in der RLR-Gruppe signifikant niedriger als in der LLR-Gruppe (4 % vs. 12 %, *p* < 0,01).

Postoperative Komplikationen traten in der RLR-Gruppe signifikant seltener auf als in der LLR-Gruppe (21 % vs. 42 %, *p* = 0,03). Schwere Komplikationen (Clavien-Dindo ≥ III) wurden in 9 % der Fälle nach RLR und in 16 % nach LLR beobachtet.

Leberspezifische Komplikationen wie ein Posthepatektomie-Leberversagen (Grad B/C) traten bei 3 % der roboterassistierten und 6 % der laparoskopischen Eingriffe auf (*p* = 0,34), eine Posthepatektomie-Gallenleckage (Grad B/C) bei 5 % vs. 8 % (*p* = 0,54) und eine Posthepatektomie-Leberblutung bei 1 % vs. 3 % (*p* = 0,57). Die mediane Krankenhausverweildauer war in beiden Gruppen vergleichbar (6 Tage [4–9] vs. 6 Tage [5–10], *p* = 0,34).

### Low/Intermediate-Leberresektionen

In der Subgruppe der Low/Intermediate-Leberresektionen (*n* = 218) wurden 193 Resektionen laparoskopisch und 25 roboterassistiert durchgeführt. Die mediane Operationszeit war in der RLR-Gruppe signifikant länger (180 min [122–237] vs. 119 min [77–180], *p* = 0,003). Die Konversionsrate war auch hier in der RLR-Gruppe geringer (4 % vs. 7 %, *p* = 0,71), jedoch ohne statistische Signifikanz. Der intraoperative Blutverlust war in beiden Gruppen gering und unterschied sich nicht signifikant (150 ml [50–300] vs. 100 ml [10–300], *p* = 0,48).

Die postoperative Komplikationsrate war in beiden Gruppen vergleichbar (12 % vs. 20 %, *p* = 0,66). Schwere Komplikationen (Clavien-Dindo ≥ III) traten in 4 % (RLR) bzw. 7 % (LLR) der Fälle auf.

Leberspezifische Komplikationen wie ein Posthepatektomie-Leberversagen (Grad B/C) wurden in beiden Gruppen nicht beobachtet. Eine Posthepatektomie-Gallenleckage (Grad B/C) trat in der LLR-Gruppe bei 4 % der Fälle auf, in der RLR-Gruppe wurde sie nicht beobachtet (*p* = 0,33). Eine Posthepatektomie-Leberblutung (Grad B/C) wurde in der RLR-Gruppe nicht beobachtet, in der LLR-Gruppe trat sie bei 1 % der Fälle auf (*p* = 0,61).

Die mediane Krankenhausverweildauer war in beiden Gruppen ebenfalls vergleichbar (4 Tage [4–5] vs. 4 Tage [3–6], *p* = 0,63).

### Jahresbezogene Entwicklung

Eine Übersicht zur jährlichen Entwicklung der Operationsmethoden und Outcome-Parameter findet sich in *Tab. 4 und 5 (Zusatzmaterial online)*.

**Abb. 2:**
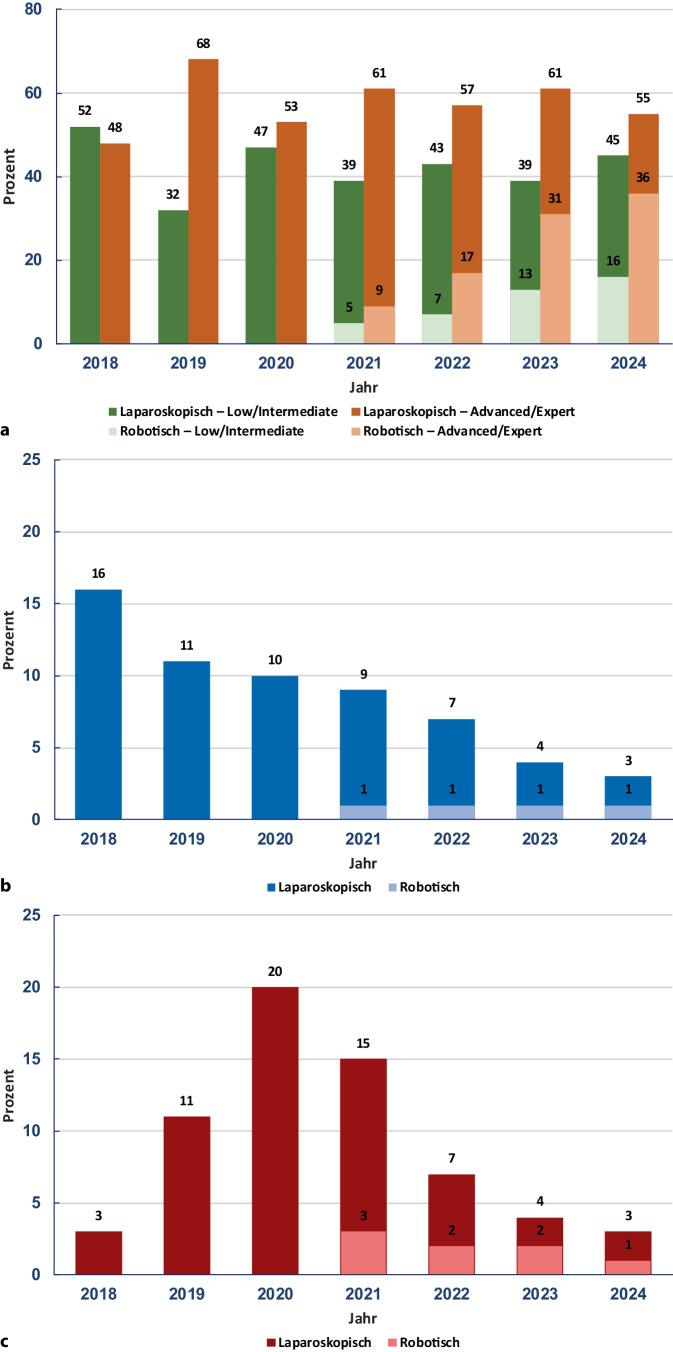
Jahresbezogene Entwicklung von Resektionsverfahren, Konversions- und Komplikationsraten (2018 bis 2024). **a** Entwicklung der jährlichen Fallzahlen gemäß der IWATE-Schwierigkeitsgrade. **b** Entwicklung der Konversionsraten. **c** Entwicklung der Rate an Clavien-Dindo-Komplikationen ≥ 3

Während in den ersten 3 Jahren des Untersuchungszeitraums ausschließlich laparoskopische Leberresektionen durchgeführt wurden, stieg der Anteil roboterassistierter Eingriffe ab dem Jahr 2021 kontinuierlich an und erreichte im Jahr 2024 bereits 53 % (Abb. 2a).

Der Anteil an Major-Resektionen nahm im Verlauf der Jahre deutlich ab – von 26–28 % in den Jahren 2018 bis 2019 auf 10–15 % in den Jahren 2023 und 2024.

Trotz einer gleichzeitigen Zunahme der Eingriffskomplexität konnte im Beobachtungszeitraum eine deutliche Reduktion schwerwiegender postoperativer Komplikationen (Clavien-Dindo ≥ III) verzeichnet werden: von 20 % im Jahr 2020 auf 4 % im Jahr 2024 (Abb. 2b). Ebenso war ein Rückgang der Konversionsrate von 16 % im Jahr 2018 auf 4 % im Jahr 2024 zu beobachten (Abb. 2c).

Zusätzlich wurde eine separate Analyse der roboterassistierten Resektionen zwischen 2021 bis 2022 und 2023 bis 2024 durchgeführt (*Zusatzmaterial online Tab. 6*). Trotz eines Anstiegs technisch anspruchsvoller Eingriffe zeigten sich keine signifikanten Unterschiede hinsichtlich Konversionsrate, Komplikationen oder Blutverlust.

Neben diesen kurzzeitigen Outcome-Parametern wurde auch die 90-Tage-Mortalität systematisch erfasst. Insgesamt verstarben 17 (3 %) Patienten innerhalb von 90 Tagen nach der Operation (Clavien-Dindo Grad V). Die zugrunde liegenden Diagnosen, Resektionsverfahren und Komplikationsverläufe sind in *Tab. 7 (Zusatzmaterial online*) zusammengefasst.

## Diskussion

Diese Studie beschreibt die Entwicklung, das operative Spektrum und die perioperativen Ergebnisse von 526 konsekutiven elektiven minimalinvasiven Leberresektionen, die zwischen 2018 und 2024 an einem High-Volume-Zentrum durchgeführt wurden. Der Fokus lag auf dem Vergleich von LLR und RLR unter Berücksichtigung des IWATE-Schwierigkeitsgrads [10]. Unsere Ergebnisse zeigen, dass beide Verfahren sicher und effektiv durchgeführt werden können, wobei sich insbesondere bei technisch anspruchsvollen Resektionen Vorteile für die roboterassistierte Technik ergeben.

Seit der Einführung der roboterassistierten Leberchirurgie im Jahr 2021 konnte der Anteil roboterassistierter Resektionen kontinuierlich gesteigert werden und betrug im Jahr 2024 bereits über 50 % aller minimalinvasiven Eingriffe. Dieser Trend spiegelt die zunehmende internationale Akzeptanz des robotischen Verfahrens wider und unterstreicht dessen erfolgreiche Integration in den klinischen Alltag [5, 7, 18]. Besonders bei Advanced/Expert-Resektionen konnte die RLR mit einer signifikant geringeren Konversionsrate, reduziertem Blutverlust und einer niedrigeren postoperativen Morbidität überzeugen. Neben den technischen Vorteilen der Robotik ist dabei zu berücksichtigen, dass in unserem Zentrum bereits vor Einführung der RLR eine breite Erfahrung in der laparoskopischen Leberchirurgie bestand. Diese etablierte Expertise hat wesentlich dazu beigetragen, dass die Lernkurve für RLR nicht bei „null“ begann, sondern viele grundlegende Aspekte der minimalinvasiven Leberchirurgie bereits routiniert beherrscht wurden. Die guten Ergebnisse unter RLR sind daher nicht allein auf das robotische System zurückzuführen, sondern auch auf die fundierte Erfahrung des chirurgischen Teams mit laparoskopischen Techniken, standardisierten Abläufen und der interdisziplinären Versorgung komplexer Leberpatienten.

Wie in vielen anderen Studien zeigte sich auch in unserer Kohorte eine signifikant längere Operationszeit bei RLR, was sich neben der längeren Vorbereitungszeit, dem Andocken des Systems und dem aufwendigeren Instrumentenwechsel wahrscheinlich v. a. durch ein insgesamt vorsichtigeres und langsameres Vorgehen im Rahmen der Lernkurve erklären lässt [7, 8, 19–21]. Dieser Zeitunterschied scheint jedoch klinisch vertretbar, wenn man die geringere Komplikationsrate berücksichtigt.

Ein häufig diskutierter möglicher Nachteil der RLR ist das potenziell erhöhte Risiko für Galleleckagen, unter anderem aufgrund limitierter Auswahl an Versiegelungs- und Dissektionsinstrumenten im robotischen System [18, 20, 22]. In unserer Kohorte zeigte sich jedoch keine erhöhte Rate an Galleleckagen nach RLR. Wir führen dies auf die in unserem Zentrum etablierte standardisierte Technik der „Scissor Hepatectomy“ zurück [17]. Diese erlaubt eine präzise Parenchymdurchtrennung unter Verwendung der robotischen Monopolarschere und bipolarer Koagulation – ohne die Notwendigkeit zusätzlicher energieunterstützter Versiegelungsinstrumente.

Ein weiterer relevanter Aspekt unserer Analyse ist die beobachtete Verschiebung hin zu parenchymsparenden Resektionen. Der Anteil an Major-Resektionen hat über den Studienzeitraum deutlich abgenommen, während atypische Resektionen sowie Mono- und Bisegmentektomien mittlerweile über 65 % aller Eingriffe ausmachen. Anatomische Major-Resektionen wurden hingegen zunehmend selektiv und seltener durchgeführt. Dieses chirurgische Prinzip zielt auf den Erhalt eines möglichst großen funktionellen Restlebervolumens ab – ein entscheidender Faktor für das postoperative Ergebnis und v. a. für das Langzeitüberleben, wie in mehreren Studien beschrieben wurde [23, 24]. Der damit einhergehende technische Mehraufwand wird bewusst in Kauf genommen, da er durch die langfristigen Vorteile für die Patienten gerechtfertigt ist. Darüber hinaus ermöglichen parenchymsparende Resektionen potenziell spätere Wiederholungseingriffe, insbesondere bei Patienten mit Tumorrezidiv oder metachronen Leberläsionen. Solche Re-Resektionen werden an unserem Zentrum ebenfalls in der Mehrzahl der Fälle sicher und effektiv minimalinvasiv durchgeführt, wie wir bereits in einer separaten Analyse zeigen konnten [14]. Im Kontext parenchymsparender Resektionsstrategien erwies sich eine Obergrenze von 4 atypischen Resektionen pro minimalinvasivem Eingriff als praktikabel und sicher. Diese Zahl stellt keinen starren Grenzwert dar, sondern basiert auf praktischen Erwägungen hinsichtlich Übersichtlichkeit, technischer Durchführbarkeit und Patientensicherheit. Bei mehr als 4 Resektionsstellen handelt es sich meist um topografisch verstreut liegende Läsionen, wodurch eine optimale Trokarplatzierung und instrumentelle Erreichbarkeit erheblich erschwert werden. Zudem steigt mit der Anzahl an Resektionsflächen das Risiko für intraoperative Komplikationen wie Blutungen oder Galleleckagen, und auch die Operationszeit verlängert sich teils erheblich. Zwar wären technisch auch mehr als 4 Einzelresektionen minimalinvasiv möglich, doch operative Zeiten von über 6–8 h bedeuten eine Belastung sowohl für das Operationsteam als auch für die Patienten. Aus diesen Gründen wurden Eingriffe mit mehr als 4 atypischen Resektionen in unserer Klinik in der Regel primär offen durchgeführt. Diese Grenze hat sich in unserem Zentrum als praxisrelevant etabliert und wird inzwischen routinemäßig in die präoperative Entscheidungsfindung einbezogen. Insgesamt trägt diese Erfahrung wesentlich zur patientenindividuellen Auswahl des Zugangswegs und zur Vermeidung unnötiger Konversionen bei.

Ein weiterer relevanter Befund unserer Analyse ist die kurze Krankenhausverweildauer mit einem Median von 6 Tagen. Damit liegen wir deutlich unter der im Fallpauschalen-Katalog des Instituts für das Entgeltsystem im Krankenhaus (InEK) hinterlegten mittleren Verweildauer für Leberresektionen, die je nach Eingriffstyp in der Regel zwischen 10 und 13 Tagen liegt [25]. Dieser Unterschied unterstreicht das Potenzial der minimalinvasiven Technik im Hinblick auf eine schnellere postoperative Erholung.

Abschließend sollte betont werden, dass die Ergebnisse dieser Studie nicht ausschließlich die chirurgische Expertise widerspiegeln, sondern auch Ausdruck einer etablierten interdisziplinären Teamstruktur, strukturierter Ausbildungswege und eines kontinuierlichen interdisziplinären Qualitätsmanagements sind – Faktoren, die für eine sichere und effiziente Umsetzung komplexer Leberchirurgie essenziell sind.

Trotz der Stärken dieser Studie – einschließlich der großen Fallzahl, standardisierter Operationstechniken und der objektiven Risikostratifizierung mittels IWATE-Scores – müssen mehrere Limitationen berücksichtigt werden. Erstens handelt es sich um eine monozentrische, retrospektive Analyse mit potenziellem Selektionsbias. Um diesen zu minimieren, wurden klar definierte Ein- und Ausschlusskriterien sowie standardisierte Definitionen für alle Variablen angewendet. Zweitens wurde die Studie an einem High-Volume-Zentrum mit großer Erfahrung in der minimalinvasiven Leberchirurgie durchgeführt. Die Übertragbarkeit der Ergebnisse auf Kliniken mit geringeren Fallzahlen oder weniger Erfahrung in der laparoskopischen und roboterassistierten Leberchirurgie ist daher möglicherweise eingeschränkt. Drittens lag der Fokus der vorliegenden Analyse auf den kurzzeitigen postoperativen Ergebnissen. Langzeitonkologische Endpunkte wie das rezidivfreie und das Gesamtüberleben waren nicht Gegenstand dieser Untersuchung und bedürfen zukünftiger prospektiver Studien. Viertens ist anzumerken, dass der Anteil roboterassistierter Resektionen (*n* = 101) lediglich rund 19 % der Gesamtfälle ausmacht. Zwar ist der Anteil in den letzten Jahren deutlich gestiegen, dennoch bleibt die absolute Fallzahl im Vergleich zur LLR-Gruppe begrenzt, was die Aussagekraft der Subgruppenanalysen potenziell einschränkt. Zukünftige Studien mit größeren Fallzahlen und prospektivem Design sind notwendig, um die beobachteten Unterschiede weiter zu bestätigen.

## Fazit für die Praxis

- Minimalinvasive Leberresektionen können an High-Volume-Zentren sicher durchgeführt werden.
- Die roboterassistierte Leberchirurgie bietet insbesondere bei komplexen Resektionen Vorteile, u. a. geringere Komplikations‑, Konversions- und Blutungsraten.
- Die Lernkurve in der robotischen Chirurgie kann durch vorhandene laparoskopische Expertise deutlich verkürzt werden.
- Der Einsatz standardisierter Techniken wie der „Scissor Hepatectomy“ führte möglicherweise zu einer vergleichsweise niedrigen Rate an Galleleckagen im Rahmen der RLR.
- Parenchymsparende Resektionstechniken ermöglichen sichere Wiederresektionen bei Tumorrezidiven.
- Im minimalinvasiven Setting hat sich eine Obergrenze von 4 atypischen Resektionen pro Eingriff als praktikabel erwiesen – wenn mehr als 4 atypische Resektionen erforderlich sind, sollte bei der präoperativen Planung frühzeitig eine offene Operation in Erwägung gezogen werden.
- Die mediane Krankenhausverweildauer betrug 6 Tage und lag damit deutlich unter der im InEK-Fallpauschalenkatalog hinterlegten mittleren Verweildauer – ein Vorteil, der die Effizienz und das Erholungspotenzial der minimalinvasiven Technik unterstreicht.
- Strukturierte interdisziplinäre Abläufe und kontinuierliches Training sind essenziell für die erfolgreiche Umsetzung.

## Supplementary Information

Zusätzliche Tabellen 4–7. Tab. 4 Jahresbezogene Patientenmerkmale der Studienkohorte; Tab. 5 Jahresbezogene Entwicklung operativer Parameter und postoperativer Ergebnisse der Studienkohorte; Tab. 6 Zeitliche Entwicklung chirurgischer Kennzahlen bei roboterassistierten Leberresektionen; Tab. 7 Charakteristika und Todesursachen der innerhalb von 90 Tagen verstorbenen Patient:innen (Clavien-Dindo Grad V)

## Data Availability

Die erhobenen Datensätze können auf begründete Anfrage in anonymisierter Form beim korrespondierenden Autor angefordert werden. Die Daten befinden sich auf einem Datenspeicher am Studienzentrum der Chirurgischen Klinik der Universitätsmedizin Mannheim.
