# Supplementary material for: 500 minimally invasive liver resections—Experiences, results and technical developments of a high-volume center
Source: Chirurgie (Heidelb). 2025 Sep 6;97(4):301–9. [Article in German] doi: 10.1007/s00104-025-02373-1 (PMC13004734; doi:10.1007/s00104-025-02373-1)
Supplement: Supplementary file 1 — Zusätzliche Tabellen 4–7. Tab. 4 Jahresbezogene Patientenmerkmale der Studienkohorte; Tab. 5 Jahresbezogene Entwicklung operativer Parameter und postoperativer Ergebnisse der Studienkohorte; Tab. 6 Zeitliche Entwicklung chirurgischer Kennzahlen bei roboterassistierten Leberresektionen; Tab. 7 Charakteristika und Todesursachen der innerhalb von 90 Tagen verstorbenen Patient:innen (Clavien-Dindo Grad V) [file 104_2025_2373_MOESM1_ESM.pdf]

Zusatzmaterial online

Tabelle 4: Jahresbezogene Patientenmerkmale der Studienkohorte

	2018<br>n= 31	2019<br>n= 85	2020<br>n= 93	2021<br>n= 98	2022<br>n= 81	2023<br>n= 71	2024<br>n= 67
**Alter, Jahre[a]**	60 (49–75)	64 (56–71)	71 (60–77)	67 (58–74)	65 (54–71)	63 (52–72)	67 (59–71)
**BMI, kg/m²[a]**	26 (23–30)	26 (23–29)	27 (24–30)	27 (23–29)	27 (23–29)	27 (22–30)	27 (23–30)
**Geschlechterverteilung (m:w)**	13: 18	56:29	52:41	28:40	46:35	37:34	31:36
**ASA-Klassifikation[b]**							
I	1 (3)	5 (6)	3 (3)49	6 (6)	5 (6)	4 (6)	2 (3)
II	19 (61)	42 (49)	48 (52)	55 (56)	42 (52)	30 (42)	20 (30)
III	10 (32)	38 (45)	41 (44)	33 (37)	34 (42)	35 (49)	42 (62)
IV	1 (3)	0 (0)	1 (1)	4 (4)	0 (0)	2 (3)	3 (4)
**Kardiovaskuläre Vorerkrankungen[b]**	17 (54)	52 (61)	62 (67)	56 (57)	40 (49)	25 (35)	34 (51)
**Diabetes mellitus[b]**	3 (10)	21 (25)	28 (30)	20 (20)	16 (18)	10 (14)	14 (21)
**Pulmonale Vorerkrankungen[b]**	5 (16)	13 (15)	13 (14)	14 (14)	14 (17)	9 (13)	14 (21)
**Leberzirrhose[b]**	1 (3)	17 (20)	13 (14)	9 (9)	10 (13)	7 (10)	10 (15)
Child A	1 (3)	14 (16)	13 (14)	8 (8)	9 (11)	5 (7)	6 (9)
Child B	0 (0)	3 (4)	0 (0)	1 (1)	1 (1)	2 (3)	4 (6)
**Vorbehandlungen[b]**							
Vorangegangene Leberresektion	4 (13)	21 (25)	18 (19)	21 (21)	13 (16)	12 (17)	13 (19)
Vorangegangene lokalregionale Therapie	0 (0)	5 (6)	2 (2)	5 (5)	3 (4)	3 (4)	1 (1)
Vorangegangene systemische Therapie	2 (6)	12 (14)	18 (19)	13 (13)	12 (15)	7 (10)	7 (10)
**Diagnose**							
**Primär maligne Lebertumore[b]**	8 (26)	34 (40)	31 (33)	40 (41)	20 (25)	19 (27)	19 (28)
HCC	5 (16)	25 (29)	18 (19)	22 (22)	15 (19)	14 (20)	12 (18)
CCC	3 (10)	7 (8)	12 (13)	17 (17)	4 (5)	4 (6)	6 (9)
GBC	0 (0)	2 (2)	1 (1)	1 (1)	1 (1)	1 (1)	1 (1)
**Lebermetastasen[b]**	14 (45)	36 (42)	48 (52)	42 (43)	34 (42)	36 (51)	22 (33)
**Benigne Lebertumore[b]**	9 (29)	15 (18)	14 (15)	16 (16)	27 (33)	16 (26)	26 (39)

BMI Körpermassenindex (body mass index); *ASA* Amerikanische Gesellschaft der Anästhesisten (American Society of Anesthesiologists); *INR* International normalisierte Ratio*;* *HCC* Hepatozelluläres Karzinom; *CCC* Cholangiozelluläres Karzinom; *GBC* Gallenblasenkarzinom

[a]Werte als Median (Interquartilsabstand)

[b]Werte als absolute Zahlen (Prozentwert)

Tabelle 5: Jahresbezogene Entwicklung operativer Parameter und postoperativer Ergebnisse der Studienkohorte

	2018 n= 31	2019 n= 85	2020 n= 93	2021 n= 98	2022 n= 81	2023 n= 71	2024 n= 67
**Resektionsausmaß[a]**							
Major Resektion	8 (26)	24 (28)	17 (18)	23 (23)	10 (12)	11 (15)	7 (10)
Minor Resektion	23 (74)	(72)	76 (82)	75 (76)	71 (88)	60 (85)	60 (89)
**Zugangsweg[a]**							
Laparoskopisch	31 (100)	85 (100)	93 (100)	84 (86)	61 (75)	40 (56)	32 (48)
Robotisch	-	-	-	14 (14)	21 (25)	31 (44)	35 (52)
**Konversion[a]**	4 (16)	9 (11)	9 (10)	10 (10)	7 (8)	4 (6)	3 (4)
**Operationsverfahren[a]**							
Atypische Leberresektion	13 (42)	27 (32)	44 (47)	45 (46)	28 (36)	27 (38)	29 (43)
(Erweiterte) Hemihepatektomie rechts	3 (10)	18 (21)	11 (12)	14 (14)	9 (11)	8 (11)	6 (9)
(Erweiterte) Hemihepatektomie links	5 (16)	6 (7)	6 (6)	9 (9)	2 (2)	3 (4)	1 (1)
Linke laterale Sektorektomie	4 (13)	5 (6)	7 (8)	6 (6)	9 (11)	7 (10)	4 (6)
Rechts posteriore Sektorektomie	0 (0)	3 (4)	5 (5)	5 (5)	0 (0)	5 (7)	0 (0)
Rechts anteriore Sektorektomie	0 (0)	0 (0)	0 (0)	1 (1)	2 (0)	3 (4)	0 (0)
Sonstige Mono- oder Bisegmentresektionen	6 (19)	31 (36)	26 (28)	29 (30)	24 (30)	20 (28)	22 (33)
**Operationszeit, Minuten[b]**	250 (138–339)	231 (145–337)	202 (110–309)	231 (130–310)	154 (91–258)	226 (140–309)	242 (119–355)
**Pringle-Manöver[a]**	7 (23)	23 (27)	42 (45)	58 (59)	32 (40)	36 (51)	35 (53)
Dauer, Minuten[b]	20 (17–34)	15 (10–29)	36 (22–57)	48 (27–67)	43 (23–72)	42 (30–88)	34 (17–66)
**Blutverlust, ml[b]**	300 (100–500)	500 (200–1200)	400 (100–1000)	400 (125–850)	200 (100–500)	350 (100–375)	300 (125–950)
**IWATE-Score[a]**							
Advanced/Expert	15 (48)	58 (68)	49 (53)	60 (61)	46 (57)	43 (61)	37 (55)
Low/Intermediate	16 (52)	27 (32)	44 (47)	38 (39)	35 (43)	28 (39)	30 (45)
**Postoperative Komplikationen[a,c]**	14 (45)	42 (49)	33 (35)	39 (40)	12 (15)	7 (10)	52 ()
Grad I	12 (39)	18 (21)	7 (8)	14 (17)	2 (2)	1 (1)	1 (1)
Grad II	1 (3)	15 (8)	7 (8)	9 (9)	2 (2)	2 (3)	3 (4)
Grad III	1 (3)	6 (7)	15 (16)	7 (7)	3 (4)	3 (4)	1 (1)
Grad IV	0 (0)	1 (1)	3 (3)	3 (3)	3 (4)	0 (0)	1 (1)
Grad V	0 (0)	2 (2)	1 (1)	6 (6)	2 (2)	1 (1)	2 (3)
**Art der Komplikationen[a]**							
Wundinfektion	1 (3)	2 (2)	1 (1)	3 (3)	2 (2)	2 (3)	1 (1)
Platzbauch	0 (0)	3 (4)	0 (0)	2 (2)	0 (0)	1 (1)	0 (0)

Pleuraerguss mit Atelektasen	0 (0)	1 (1)	4 (4)	12 (12)	3 (4)	2 (3)	2 (3)
Lungenembolie	0 (0)	1 (1)	2 (2)	2 (2)	0 (0)	1 (1)	2 (3)
Posthepatektomie-Leberblutung[d]	0 (0)	2 (2)	2 (2)	4 (4)	0 (0)	0 (0)	0 (0)
Posthepatektomie Gallenleckage[d]	1 (3)	2 (2)	7 (7)	9 (9)	5 (6)	2 (3)	3 (4)
Posthepatektomie-Leberversagen[d]	1 (3)	4 (5)	2 (2)	2 (2)	4 (6)	2 (3)	0 (0)
**Krankenhausverweildauer, Tage[b]**	5 (4–7)	6 (4–8)	6 (4–9)	6 (4–9)	4 (3–6)	5 (4–7)	5 (3–8)

[a]Werte als absolute Zahlen (Prozentwert)
[b]Werte als Median (Interquartilsabstand)
[c]Klassifikation nach Clavien-Dindo
[d]Klassifikation gemäß ISGLS; ausschließlich Grad B und C berücksichtigt

Tabelle 6: Zeitliche Entwicklung chirurgischer Kennzahlen bei roboterassistierten Leberresektionen

	**2021-2022**<br>**n = 35**	**2022-2023**<br>**n = 66**	***p*-Wert**
**Resektionsausmaß**[a]			0.32
Atypische Leberresektion	12 (34)	20 (30)	
(Erweiterte) Hemihepatektomie rechts	7 (20)	10 (15)	
(Erweiterte) Hemihepatektomie links	5 (14)	2 (3)	
Linke laterale Sektorektomie	4 (11)	4 (6)	
Rechts posteriore Sektorektomie	1 (3)	3 (5)	
Rechts anteriore Sektorektomie	3 (9)	2 (3)	
Sonstige Mono- oder Bisegmentresektionen	5 (14)	26 (39)	
**Konversion**[a]	2 (6)	2 (3)	0.42
**Operationszeit, Minuten**[b]	287 (178-400)	258 (200-380)	0.62
**Blutverlust, ml**[b]	350 (100-800)	400 (200-900)	0.74
**IWATE-Score**[a]			0.19
Advanced/Expert	23 (66)	53 (80)	
Low/Intermediate	12 (34)	13 (20)	
**Postoperative Komplikationen**[a,c]	11 ()	8 ()	0.14
Grad I	4	1	
Grad II	2	4	
Grad III	2	1	
Grad IV	1	0	
Grad V	2	2	
**Art der Komplikationen**[a]			
Posthepatektomie-Leberblutung[d]	1	0	0.20
Posthepatektomie Gallenleckage[d]	2	2	0.62
Posthepatektomie-Leberversagen[d]	0	2	0.27
**Krankenhausverweildauer, Tage**[c]	5 (4-7)	5 (4-8)	0.31

[a]Werte als absolute Zahlen (Prozentwert)
[b]Werte als Median (Interquartilsabstand)
[c]Klassifikation nach Clavien-Dindo
[d]Klassifikation gemäß ISGLS; ausschließlich Grad B und C berücksichtigt

Tabelle 7: Charakteristika und Todesursachen der innerhalb von 90 Tagen verstorbenen Patient:innen (Clavien-Dindo Grad V)

Geschlecht/ Alter	ASA	Leberzirrhose[a]	Diagnose	Resektionsverfahren	Todesursache / Komplikationskaskade[b]
M 63	ASA III	-	Bilobör metastasiertes Rektumkarzinom	Erweiterte Hemihepatektomie rechts bei Z.n. In-Situ-Split und Pfortaderembolisation	PHLF Grad C → PHH Grad C → Kolonischämie → Septischer Schock → Multiorganversagen → Exitus letales
M 62	ASA III	Child A	Cholangiocelluläres Karzinom	Hemihepatektomie rechts	PHLF Grad B → PHH Grad C → Multiple superinfizierte Verhalte → Septischer Schock → Multiorganversagen → Exitus letales
W 72	ASA III	Child A	Cholangiocelluläres Karzinom	Hemihepatektomie rechts	PHBL Grad B → PHLF Grad C → Multiorganversagen → Exitus letales
M 87	ASA III	Child A	Hepatocelluläres Karzinom	Mesohepatektomie (Segment IV, V, VIII)	PHLF Grad C → PHH Grad C → Nosokomiale Pneumonie → ARDS → Exitus letales
W 62	ASA III	-	Cholangiocelluläres Karzinom	Hemihepatektomie links, Pfortaderresektion und -rekontruktion als E-E-Anastomose, Biliodigestive Anastomose	PHBL Grad B → PHLF Grad C → PHH Grad C → Multiorganversagen → Exitus letales
M 82	ASA III	Child A	Hepatocelluläres Karzinom	Hemihepatektomie rechts bei Z.n. Pfortaderembolisation rechts	PHBL Grad C → Sepsis → Kolonischämie → Dünndarmischämie → Exitus letales
M 62	ASA III	Child B	Hepatocelluläres Karzinom	Laparoskopisch-auf-offen konvertierte Bisegmentektomie (Segment 4a + 8)	PHH Grad C → Kolonischämie → Septischer Schock → Multiorganversagen → Exitus letales
M 63	ASA III	Child A	Hepatocelluläres Karzinom	Hemihepatektomie rechts	PHLF Grad C → Kolonischämie → Septischer Schock → Multiorganversagen → Exitus letales
M 71	ASA III	-	Cholangiocelluläres Karzinom	Laparoskopisch-auf-offen konvertierte Hemihepatektomie links	PHLF Grad C → PHBL Grad B → Kolonischämie → Septischer Schock → Multiorganversagen → Exitus letales
W 83	ASA III	-	Cholangiocelluläres Karzinom	Anatomische Lebersegment-IV-Resektion	Postoperative Lungenarterienembolie
M 74	ASA III	-	Kolorektale Lebermetatsasen	Hemihepatektomie links	Fulminanter Myokardinfarkt → frustrane Reanimation → Exitus letales
W 56	ASA III	Child A	Hepatocelluläres Karzinom	Anatomische Lebersegment-VIII-Resektion bei Z.n. linkslateraler Resektion	PHLF Grad C → Kolonischämie → Septischer Schock → Multiorganversagen → Exitus letales
W 67	ASA III	-	Cholangiocelluläres Karzinom	Erweiterte Hemihepatektomie links	Myokardperforation bei iatrogener Myokardverletzung
M 79	ASA III	-	Angiosarkom der Leber	Mesohepatektomie (Segment IV, V, VIII)	PHLF Grad C → Nosokomale Pneumonie → Multiorganversagen → Exitus letales
M 63	ASA III	-	Angiosarkom der Leber	Anatomische Lebersegment-II-Resektion	Postoperative Cholangitis und Cholestase → Magenentleerungsstörung → Rascher Tumorprogress und Basistherapie
M 77	ASA III	Child A	Hepatocelluläres Karzinom	Hemihepatektomie rechts	PHLF Grad C → PHH Grad C → Multiple intraabdominelle Verhalte → Septischer Schock → Multiorganversagen → Exitus letales

W 72	ASA III	Child A	Cholangiocelluläres Karzinom	Hemihepatektomie rechts	PHBL Grad B → PHLF Grad C → Multiorganversagen → Exitus letales

ASA Amerikanische Gesellschaft der Anästhesisten (American Society of Anesthesiologists); *M* männlich; *W* weiblich; *PHLF* Posthepatektomie-Leberversagen; *PHBL* Posthepatektomie-Gallenleckage; *PHH* Posthepatektomie-Leberblutung

[a]Klassifikation nach den Child-Pugh-Kriterien

[b]Klassifikation gemäß ISGLS
